# Environmental and cost benefits of co-digesting food waste at wastewater treatment facilities

**DOI:** 10.2166/wst.2020.104

**Published:** 2020-07

**Authors:** Ben Morelli, Sarah Cashman, Xin (Cissy) Ma, Jason Turgeon, Sam Arden, Jay Garland

**Affiliations:** Eastern Research Group, 110 Hartwell Ave., Lexington, MA 02421, USA; Eastern Research Group, 110 Hartwell Ave., Lexington, MA 02421, USA; United States Environmental Protection Agency, Center for Environmental Solutions and Emergency Response, Water Infrastructure Division, 26 West Martin Luther King Drive, Cincinnati, OH 45268, USA; United States Environmental Protection Agency, Region 1, 5 Post Office Square, Suite 100, OEP 5-2, Boston, MA 02109, USA; Eastern Research Group, 110 Hartwell Ave., Lexington, MA 02421, USA; United States Environmental Protection Agency, Center for Environmental Solutions and Emergency Response, Immediate Office 26 West Martin Luther King Drive, Cincinnati, OH 45268, USA

**Keywords:** anaerobic digestion, biogas, co-digestion, LCA, resource recovery, wastewater treatment

## Abstract

The wastewater industry is undergoing a paradigm shift from focusing solely on treatment to incorporating concepts aimed at mitigating environmental impacts such as energy and nutrient recovery and water reuse. This study uses life cycle assessment and life cycle cost analysis to investigate the effect of expanding anaerobic digestion (AD) capacity and adding combined heat and power on environmental and cost indicators at a mid-sized wastewater treatment facility (WWTF) in Massachusetts, USA. Since 2014, Massachusetts has banned the disposal of organic waste from commercial organizations producing more than one ton of material per week. The WWTF’s additional digester capacity allows the co-digestion of municipal solids with a food-based engineered bioslurry due to this ban. Study data were compiled for several AD feedstock quantity and performance scenarios, and compared to a baseline scenario representative of historic plant operations prior to co-digestion. Reductions in environmental impact are demonstrated for six of eight environmental impacts, including global climate change potential and cumulative energy demand. Eutrophication potential increases by 10 percent and 24 percent across assessed scenarios. Water use remains relatively constant across scenarios. Facility energy production increases dramatically with co-digestion, satisfying 100 percent of the WWTF’s thermal energy requirement and producing surplus electricity assuming full AD capacity utilization.

## INTRODUCTION

Municipal waste service providers are under continual pressure to improve their economic and environmental efficiency. Historically, these services, including wastewater treatment and solid waste disposal, have operated under a single pass framework requiring large inputs of energy, materials, and land to treat and dispose of liquid and solid waste streams while recovering little, if any, of the streams’ nutrient or energy content (Tarr 1996; Ma *et al.* 2015). In the USA for example, nearly 1 percent of all electricity consumption is attributable to municipal wastewater treatment facilities (WWTF), with up to 80 percent of that allocated to aeration and sludge processing (Electric Power Research Institute, EPRI 2013), despite the fact that the wastewater itself contains 5–10 times as much energy as is required to treat it (McCarty *et al.* 2011). On the solid waste side, anaerobic digestion (AD) of the food waste generated in the USA could power 4.5 million households (Becker *et al.* 2017); unfortunately, food waste is predominantly disposed of in landfills (United States Environmental Protection Agency, US EPA 2018) or incinerated in waste-to-energy facilities (WTE). Landfills are the third largest source of anthropogenic methane emissions in the USA, accounting for over three percent of national greenhouse gas emissions (US EPA 2019). By diverting food waste from landfills to digesters at existing WWTF, anaerobic co-digestion (AcD) of municipal sludge and food waste can generate energy (McCarty *et al.* 2011), reduce solid waste volumes, and recover valuable nutrients (Guest *et al.* 2009).

AD is not new, and has been used in the wastewater treatment and agricultural sectors for over 100 years (Mata-Alvarez *et al.* 2011; Hindle 2013). The recent attraction toward AcD has been motivated by a combination of technical, regulatory, and economic factors, including: (1) ample gains in biogas production supported by laboratory testing (Prabhu & Mutnuri 2016; Zahan *et al.* 2016), case studies (Mattioli *et al.* 2017) or both (Koch *et al.* 2016; Lensch *et al.* 2016); (2) disposal limitations, including organic waste bans to municipal solid waste facilities (Edwards *et al.* 2015; Leib *et al.* 2019); and (3) development of alternative energy markets creating opportunities for WWTF to valorize waste streams (Edwards *et al.* 2015; Nghiem *et al.* 2017).

Despite the benefits of AcD, it is increasingly acknowledged that the sustainability of waste management and disposal options go beyond the facilities themselves (Lundin *et al.* 2000; Hester & Little 2013), extending from land, air, and water impacts of upstream supply chains (Corominas *et al.* 2013) through to use of final, treated effluent (Muñoz *et al.* 2009). The complexities of the systems involved and the extent of potential impacts necessitate evaluation with system-based tools and integrated frameworks such as life cycle assessment (LCA) and life cycle cost analysis (LCCA) to measure trade-offs and identify optimal solutions (Xue *et al.* 2015).

This study aims to address these complexities and provide guidance for future investments through a comprehensive economic and environmental evaluation of an existing WWTF practicing AcD. The Greater Lawrence Sanitary District (GLSD) WWTF is a 197,000 m^3^/day (52 million gallons per day) capacity plant in Massachusetts, USA that on average treats 89,000 m^3^/day of municipal sewage and septic waste for several communities. Motivated by the recent ban on organic material disposal from commercial organizations (Commonwealth of Massachusetts 2017a), the WWTF has undergone a series of renovations to increase its digester capacity and expand its energy recovery capabilities. LCA and LCCA tools were used to (1) evaluate the baseline environmental benefits and burdens of GLSD’s wastewater treatment with AD, (2) quantify the comparative environmental differences associated with expanded AD capacity for food waste co-digestion with energy recovery, (3) determine the life cycle costs associated with the baseline and upgraded WWTF over a 30-year timespan, and (4) expand the system boundaries to understand the environmental implications of avoided food waste treatment when scaling up AcD capacity.

## METHODS

This study presents the results of a combined LCA and LCCA for a WWTF that is expanding its AcD capacity. The analysis complies with the guidelines established for conducting an LCA study in the International Organization for Standardization’s 14040 and 14044 standards (ISO 2006a, 2006b). The LCCA results were generated using net present value (NPV) methods developed by the National Institute of Standards and Technology (Fuller & Petersen 1996).

### Goal and scope definition

**Figure 1 |:**
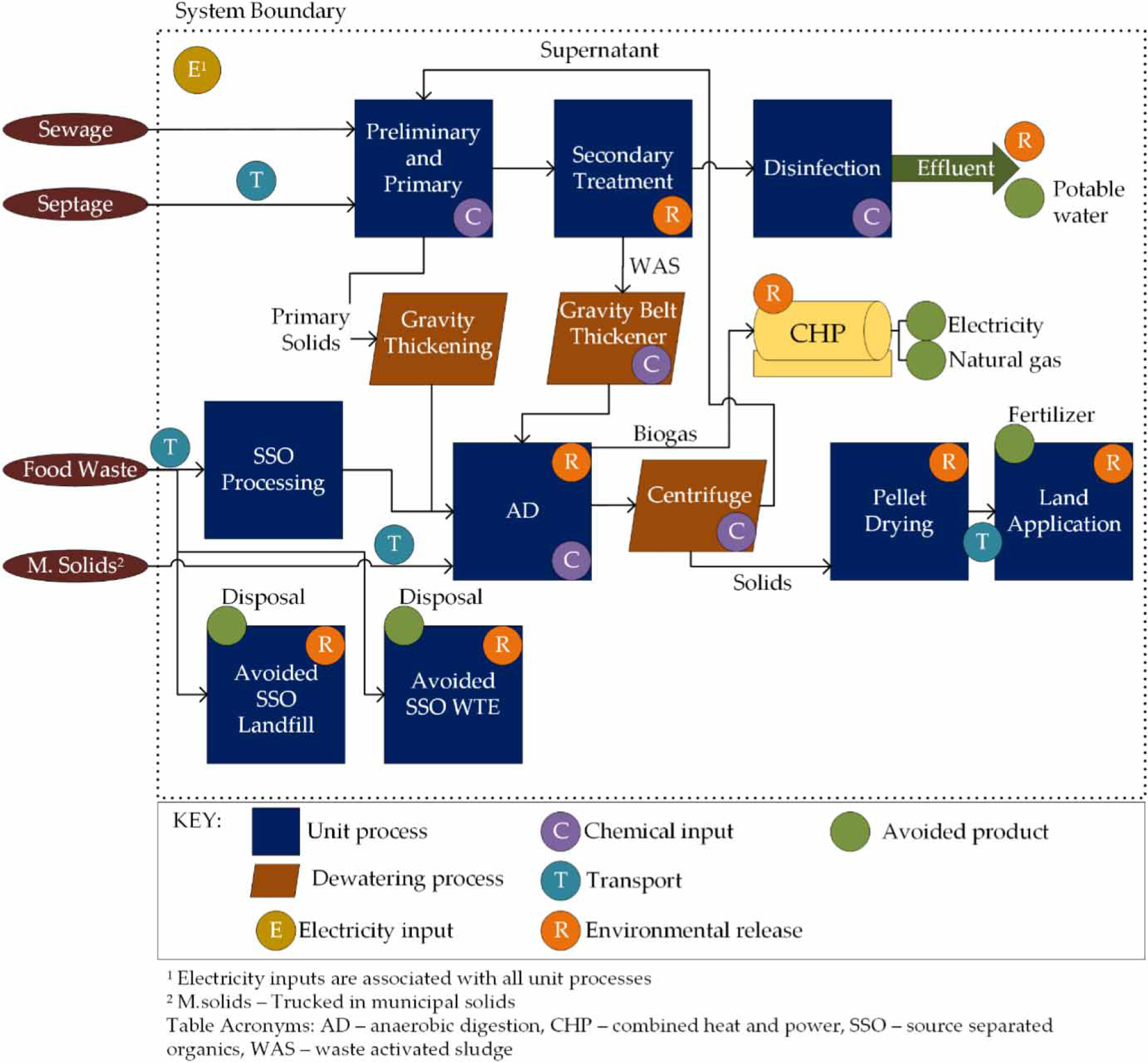
System diagram of the GLSD WWTF showing the newly installed combined heat and power system.

The goal of this study was to analyze the effect of expanding AD capacity for co-digestion of source separated organic (SSO) waste on environmental and economic sustainability indicators at the GLSD WWTF in Massachusetts. SSO is an engineered feedstock, composed primarily of industrial and institutional food waste. The feedstock is provided to the WWTF by a private waste management company, which blends and screens waste from the Greater Boston region providing a clean and consistent product for digestion. The system boundaries incorporate the historical treatment of the food waste in Massachusetts prior to the ban on commercial organic waste disposal, which was 32 percent to landfill and 68 percent to WTE incineration (Fischer 2017). A scenario analysis was used to determine the energy recovery potential of AD for two SSO acceptance scenarios and two levels of AD performance. The study’s functional unit is the treatment of one cubic meter (m^3^) of municipal wastewater with quality characteristics as defined in Table S1 (Supplementary Materials, available online). The acceptance of SSO material has a negligible effect on the volume of waste treated by the facility and was therefore excluded from the definition of the functional unit. Figure 1 shows a simplified diagram of the WWTF, including the primary inputs and outputs and the system boundary.

Preliminary and primary treatment includes the influent pump station, grit removal, bar screens and primary clarification. Primary solids are dewatered in gravity thickening units. Primary clarifier effluent proceeds to an anoxic zone that is operated to minimize nitrification in the aeration basin. The treatment plant is not operated for nutrient removal. Wastewater leaving the aeration basin is clarified prior to disinfection and dechlorination using sodium hypochlorite and sodium bisulfite, respectively. A gravity belt thickener, aided by chemical polymers, is used to dewater waste activated sludge. Effluent is discharged into the Merrimack River, approximately 30 miles (48 km) upstream of the Atlantic Ocean. The plant reuses approximately 13 percent of treated effluent, which receives an avoided burden credit for avoiding potable water production and distribution. Process-based greenhouse gas emissions result from the aeration basin and effluent discharge.

The WWTF first installed AD in 2002 and has been using the biogas in a glycol boiler to provide AD unit heat since that time. Biogas is also directly combusted in an onsite biosolids drying facility that accepts dewatered solids from the centrifuge, dries them, and pelletizes them to produce a class A exceptional quality agricultural amendment. Trucked-in municipal solids are fed directly into the AD unit. As of 2018, the plant was in the final stages of installing a fourth AD tank and a combined heat and power (CHP) system. The additional AD capacity is being used to accept SSO from regional institutional and industrial sources. The glycol boiler is being phased out in favor of the CHP system.

Two co-digestion feedstock scenarios were compared to baseline (historical) WWTF environmental impacts per cubic meter of treated wastewater. The baseline feedstock scenario is representative of conditions at GLSD prior to accepting SSO. The partial capacity feedstock scenario is representative of the WWTF using 50 percent of the available AcD capacity. Table 1 lists the quantities of waste processed in each feedstock scenario.

Each feedstock scenario was evaluated according to base (expected) and low AD performance. Anaerobic digester scenarios are defined by the five sets of parameter values listed in Table 2. All scenario results were generated using avoided end-of-life disposal pathways typical of current, Massachusetts municipal solid waste disposal. A sensitivity analysis was carried out to help understand how results may change when considering national average avoided disposal processes and hypothetical scenarios where 100 percent of food waste is diverted from landfills or WTE facilities. In the national average disposal scenario, 82 percent of food waste is disposed of in landfills, with the remaining 18 percent combusted in WTE facilities (USA EPA 2014).

### Inventory analysis

Tables 3 and 4 detail the life cycle inventory (LCI) modeled for liquid and solid treatment processes at the WWTF, respectively. Values that remain constant across scenarios are not expected to change considerably in response to estimated process changes. For example, the electricity demand of the primary clarifier is not expected to increase notably because SSO is pumped directly into the AD tanks. As a result, the clarifier sees negligible increases in the volume of water and solids processed.

Inventory data for the baseline scenario were primarily provided by GLSD staff for 2016. Inventory values based on plant records are identified by a star (★) in the LCI tables. Plant electricity consumption, based on utility records, was allocated to individual treatment processes based on data reported in a plant energy efficiency evaluation (PES & UTS 2009). The WWTF reuses a fraction of treated effluent onsite and through a program with a local WTE facility. Avoided potable water consumption from wastewater reuse provides a consistent environmental benefit across scenarios.

A model of the WWTF was developed in GPS-X (Hydromantis 2017) and validated against plant data available for the baseline scenario. The baseline GPS-X model was adjusted to reflect the increased AD capacity and feedstock inputs that correspond to the partial and full capacity scenarios. GPS-X output was used to estimate nutrient and biological oxygen demand (BOD) concentrations in secondary treatment, biogas production, and the quantity of solids sent to the centrifuge (diamond (♦)). Pollutant concentrations in plant effluent were based on plant records in the baseline scenario. Percentage removal of wastewater constituents, as calculated for the baseline scenario, was applied to increased constituent load estimates in the co-digestion scenarios to estimate effluent quality. The facility’s use of potassium permanganate (odor control), sodium hypochlorite, and sodium bisulfite are not expected to change considerably, and remain constant across scenarios.

Ferric chloride use was scaled based on the additional quantity of SSO processed, assuming similar chemical consumption per unit volume. Increased polymer consumption for the partial and full capacity scenarios was estimated using dosage rates of 0.0195 and 0.005 kg polymer/kg dry solids processed for the centrifuge and gravity belt thickener, respectively. Scaled chemical, energy, and material LCI values are marked with a hollow circle (○) in the LCI tables.

SSO processing requires water, electricity, and transportation inputs, and are identified by an asterisk (*) in the LCI tables. A 25-km transportation distance was assumed for the food waste collection route to SSO processing. The electricity consumption for SSO processing was modeled with a power requirement of 1.1 kW for a grinder capable of processing 200 kg of food waste per hour. Additional water was added to the LCI to reduce the solids content of food waste from 31 percent (RTI International 2012) to the specified 13 percent for finished bioslurry.

Nutrient and BOD concentrations in the aeration basin and facility effluent, as predicted by GPS-X, were used as the basis of process greenhouse gas estimations described in Section S2 (Supplementary Materials). Increased aeration electricity demand was based on the increase in BOD concentration in the partial and full capacity feedstock scenarios, respectively. Increased nitrogen loading to the secondary treatment process was assumed not to increase plant electricity demand as the facility operates to avoid nitrification.

Produced biogas was allocated to specific combustion units based on a hierarchy of prescribed uses and losses and marked by a plus (✚) symbol in the LCI tables. Five percent of produced biogas was assumed to be lost as fugitive emissions from the AD tanks (United Nations Framework Convention on Climate Change 2017). Of the available biogas, 10 or 20 percent was assumed to be flared, depending on the AD scenario, due to mismatches between the timing of production and consumption, CHP down-time, and a lack of storage capacity. Use of biogas for pellet drying was prioritized. Once pellet drying demand is satisfied, all remaining biogas is sent to the CHP engine. Combustion emissions for the flare, pellet drier, glycol boiler, and CHP engine were calculated based on data in the facilities air permit application as reported in Table S2 (Cousens 2016).

Increased energy demand in the pellet drying facility associated with co-digestion feedstock scenarios was estimated using biosolids production estimates from GPS-X. The pellet drying facility requires approximately 385 kWh and 9,300 MJ per metric ton of dry solids processed (CDM Smith 2013). Pelletized biosolids have final moisture, nitrogen, and phosphorus contents of 2.5, 4, and 2 percent, respectively. Pelletized biosolids are shipped across the state, approximately 121 km (75 miles) and are land applied, replacing the need for chemical fertilizers.

A fertilizer replacement value of 55 percent was applied to pellet nitrogen content, assuming that this level of mineralization occurs over a 3-year period (Smith & Durham 2002; Rigby *et al.* 2016). A phosphorus fertilizer replacement value of 95 percent was assumed (Boldrin *et al.* 2009). Urea nitrogen and single superphosphate were used as the avoided fertilizer products, assuming 46 percent nitrogen and 21 percent phosphorus as phosphorous pentoxide (P_2_O_5_) content in each avoided product, respectively. Diesel consumption for material handling and spreading was estimated assuming 1.06 liters per metric ton of material (Recycled Organic Unit 2007). The potential net increase in field emissions of ammonia and nitrous oxide (to air) and phosphorus and nitrate (to soil) were included in the LCI as described in Section S4. Biogenic carbon sequestration due to pellet land application was estimated assuming a carbon to nitrogen ratio of 7:1 (Parnaudeau *et al.* 2004; Rigby *et al.* 2016) and a sequestration factor that assumes 9 percent of carbon initially land applied remains in the soil after 100 years (i.e. is sequestered) (Favoino & Hogg 2008; Boldrin *et al.* 2009). Land application LCI values are marked with a solid square (▪) in Table 4.

Major infrastructure materials were only incorporated for the AD expansion project, including concrete, steel, gravel, and excavation, based on unit dimensions reported in the project’s energy feasibility study (CDM Smith 2013) and amortized over their useful lifespan. Building infrastructure required for the biogas cleaning and CHP system was included using the ‘building, multi-story’ Ecoinvent 2.2 unit process (Ecoinvent 2010) adapted to the USA context by substituting inputs from the USA LCI database (National Renewable Energy Laboratory 2012) and USA electrical grid data. Previously existing plant infrastructure was excluded from the analysis given that most structures are greater than 30 years old. Infrastructure LCI values are marked with a hollow square (□) in the LCI tables.

Determination of avoided landfill versus avoided WTE disposal of SSO food waste was based on the fraction of municipal solid waste that is subject to each end-of-life disposal route in Massachusetts and is marked with a filled circle (●) in the LCI tables. As of 2016, approximately 32 percent of Massachusetts municipal solid waste was disposed of in landfills, while the remainder (68 percent) was combusted at WTE facilities (Fischer 2017). Other minor disposal routes were not considered. LCI data associated with landfill and WTE facility operation were generated using EPA’s Municipal Solid Waste Decision Support Tool (RTI International 2012) and are documented in Table S5.

### Life cycle impact assessment

Per ISO standard 14040, (ISO 2006a) an LCA aims to be comprehensive in its selection of impact categories to identify potential trade-offs that may exist between impact categories. Below are listed all the impact and inventory categories that results were generated for as a part of this analysis. The LCI model was built and impact results were calculated using openLCA version 1.7.4 (GreenDelta 2018).

- Acidification potential (AP) (Bare 2012; Bare 2011).
- Eutrophication potential (EP) (Bare 2012; Bare 2011).
- Fossil fuel depletion potential (FDP) (Bare 2012; IPCC 2007).
- Global climate change potential (GCCP) (Bare 2012; Bare 2011).
- Particulate matter formation potential (PMFP) (Bare 2012; Bare 2011).
- Smog formation potential (SFP) (Bare 2012; Bare 2011).
- Cumulative energy demand (CED) (Althaus *et al.* 2010).
- Water use (WU) (Goedkoop *et al.* 2009).

### LCCA

A corresponding LCCA was carried out for each of the five feedstock-AD scenarios, allowing comparison between baseline NPV and system cost scenarios following the AD expansion and CHP project. NPV was calculated over a 30-year period using equation 5.1 in Fuller & Petersen (1996). A discounted payback period was calculated for each co-digestion and AD performance scenario to provide an indicator of the economic returns associated with AD expansion, SSO acceptance, and the installation of CHP.

Plant budget data for 2016 were the primary source of information for the LCCA. The 2016 budget provides a detailed description of annual costs associated with baseline WWTF operation. Annual costs were categorized as operation costs, material costs, chemical costs, energy costs, and plant revenue. The GLSD WWTF covers the cost of capital upgrades and non-routine maintenance projects through an annual capital expenditures budget and via loans. The facility provided estimated debt service over a 25-year time horizon, including interest and fees (Table S6). The average annual expenditure over the period from 2015 to 2017 was set as the capital expenditure budget for the duration of the cost analysis.

According to National Institute of Standards and Technology guidelines, the LCCA only applies escalation factors beyond the standard inflation rate to energy inputs (Fuller & Petersen 1996). The LCCA was performed in constant (non-inflated) dollars and uses a real discount rate corresponding to the constant dollar method. Electricity and natural gas costs were escalated according to 2017 energy escalation factors for the Northeastern USA (Lavappa *et al.* 2017; Table S7).

System NPV was calculated for both a low and base cost scenario to estimate a reasonable NPV range for each LCA scenario. Cost parameter values associated with the low and base cost scenarios are provided in Table S8. The main parameters that vary between the two cost scenarios modeled are the discount rate, electricity revenue, renewable energy credit, alternative energy credit, natural gas cost, and the SSO tipping fee.

## RESULTS AND DISCUSSION

**Figure 2 |:**
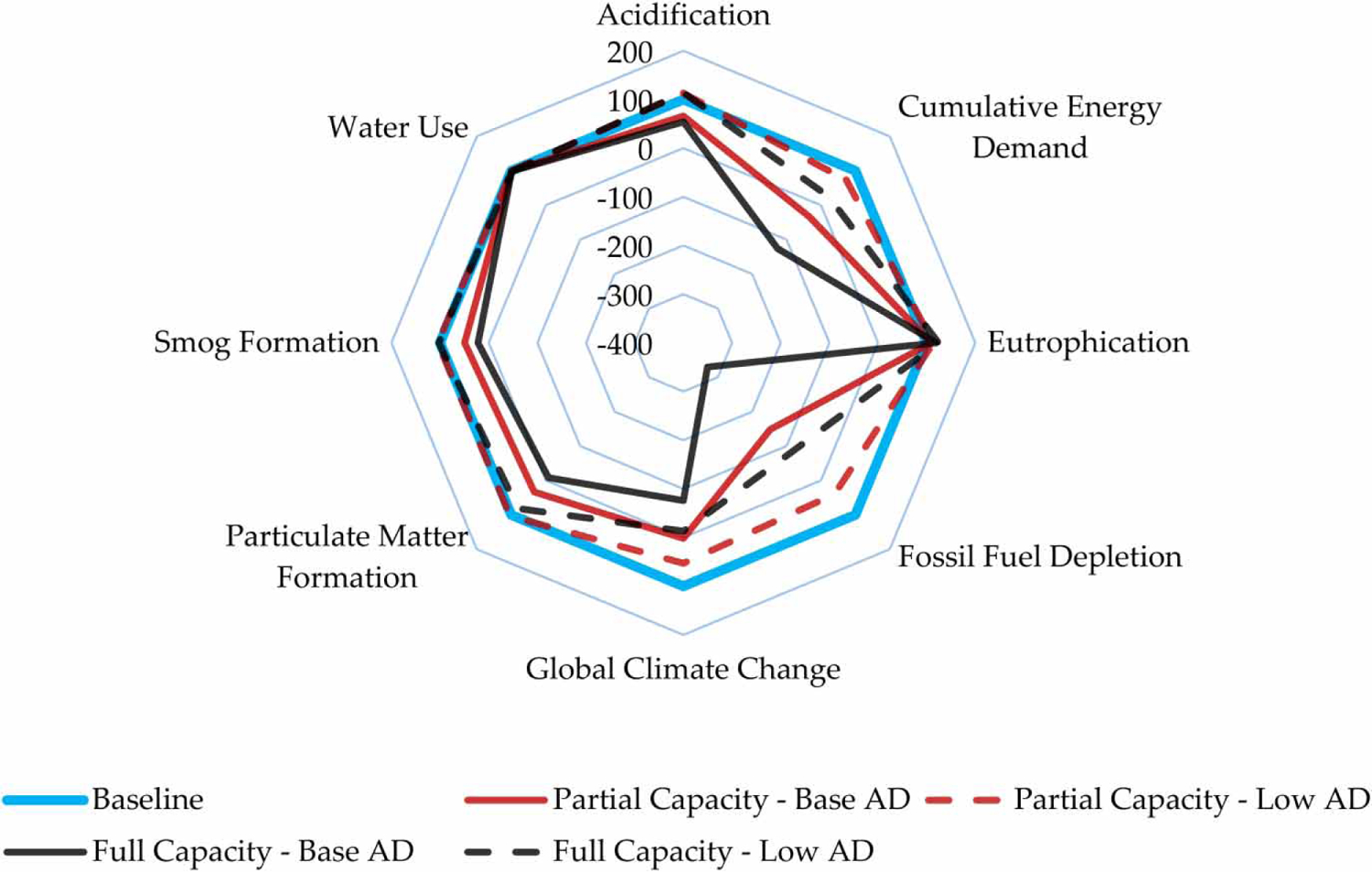
Radial plot of LCA results presented relative to the baseline scenario. [Relative impact = (scenario impact/baseline impact)*100].

Analysis results are compared against the baseline scenario, which represents historic performance of the WWTF prior to the AD and CHP expansion project. The radial plot in Figure 2 presents impact results for all categories relative to the baseline scenario, which was standardized to equal 100 (light gray line (blue in online version)). Dashed lines and solid lines represent results for the low AD and base AD performance scenarios, respectively. Values greater than 100 represent an increase in environmental impact relative to the baseline. Values between 0 and 100 represent relative reductions in impact, while values less than 0 indicate an environmental benefit.

AP, SFP, and PMFP demonstrate only marginal responses to co-digestion in the low AD performance scenario where simultaneous increases in energy production and consumption tend to cancel out in an assessment of net impact. The base AD performance scenario leads to relative reductions in impact between 46 and 108 percent in these impact categories in the full capacity scenario when 100 percent of the expanded AD capacity is being utilized for SSO co-digestion and energy production.

CED, FDP, and GCCP are the three impact categories with the greatest relative impact reduction potential, particularly within the base AD performance scenario. Reduced FDP is a direct result of substituting the use of renewable biogas for onsite natural gas combustion and fossil fuels associated with the regional New England electrical grid mix. FDP decreases sharply in the base AD scenario yielding net negative FDP impacts for both the partial and full capacity scenarios. In the case of FDP, the full capacity-base AD performance scenario yields an FDP benefit that is three times greater than the impact of the baseline scenario.

Only EP registers an increase in impact across all feedstock-AD performance scenarios due to the increased quantity of nutrients entering the WWTF associated with SSO. Eutrophication impact has the potential to increase between 10 and 24 percent depending on the SSO scenario and the fraction of nutrients that return to the primary and secondary treatment processes. Approximately 75 percent of the EP increase is attributable to increased effluent emissions in the full capacity-base AD performance scenario. Seventeen percent of the EP increase is associated with the land application of additional biosolids, and the remaining 6 percent of the increase comes from the upstream supply-chain. Additional nitrogen and phosphorus in treatment plant effluent was determined using GPS-X and assumes no additional changes to the operation of the WWTF. Since the time this study was initiated, the WWTF has begun accepting SSO material for co-digestion. Effluent monitoring data, presented in Section S7, do not yet indicate a significant increase in the release of nitrogen and phosphorus to the Merrimack River, indicating that model results may overestimate the fraction of nutrients in SSO material that returns to the primary and secondary treatment processes with biosolids centrate. These monitoring data capture SSO acceptance at a rate that is 4.5 times less than the anticipated full capacity scenario, so the actual nutrient effluent results operating at full capacity are still unknown. Water use changes very little across the scenarios, as it is almost exclusively driven by the moderate amount (13 percent) of internal and industrial effluent reuse that remains constant across scenarios.

Table 5 lists the impact results in the units designated for each impact category and the percentage change in impact results relative to the baseline scenario. Table S10 provides LCA results for the Massachusetts disposal mix scenario broken out according to treatment process contributions to environmental impact.

Co-digestion of SSO results in a minimum CED reduction of 27 percent in the partial capacity–low AD performance scenario. In the full capacity–base AD performance scenario, the facility becomes a net energy producer, with a CED impact of −6.4 MJ per m^3^ of wastewater treated. Avoided natural gas production and combustion was only included for the portion of biogas thermal energy that was put to productive use within the WWTF. Approximately 28 and 52 percent of produced thermal energy was estimated to go unused in the partial and full capacity–base AD scenarios, respectively. Table 6 summarizes the balance of energy production and consumption at the GLSD WWTF for each of the five LCA scenarios. Prior to the AD expansion and CHP installation, approximately 80 percent of the facility’s heat demand was generated using biogas. The newly installed CHP system can provide most of the facility’s direct energy consumption requirements for all feedstock-AD performance scenarios. The partial capacity–low AD performance scenario is the only scenario that is not able to completely satisfy the facilities heat demand. The full capacity–base AD performance scenario turns the GLSD WWTF into a true Water Resources Recovery Facility, not only making it energy self-sufficient but also supplying the local electrical grid with 6 Gigawatt hours of surplus electricity production per year. This energy independence also shields the facility from the energy market turbulence and increases its resilience and security in the long run. Overall, the facility can put between 71 and 81 percent of produced biogas energy content to productive use.

**Figure 3 |:**
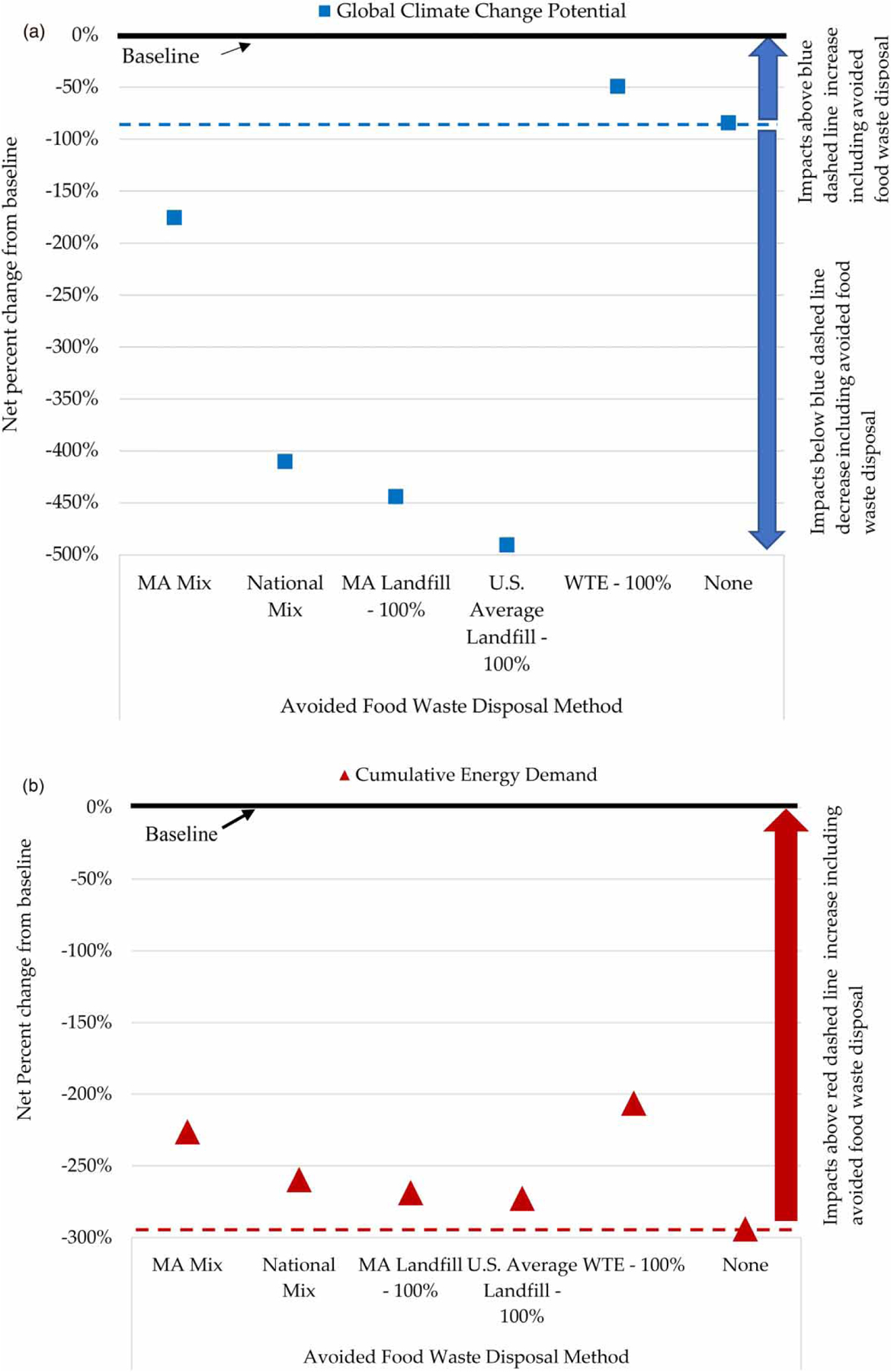
Sensitivity of GCCP (panel a) and CED (panel b) impact to avoided end-of-life disposal processes. Results for ‘None’ indicate that avoided food waste disposal is outside the scope. Impacts are shown as a percentage change relative to baseline facility operation prior to expansion for co-digestion.

Results presented above for the partial and full capacity scenarios include the net benefits and burdens of avoided waste disposal processes for discarded food, which is the basis of SSO waste. Avoided disposal process selection was based on expected Massachusetts food waste disposal routes in 2016 (Massachusetts disposal mix). Figure 3 presents sensitivity results demonstrating the effect of end-of-life disposal assumptions on CED and GCCP results. The figure compares the Massachusetts disposal mix (68 percent to WTE incineration and 32 percent to landfill) against the national disposal mix and sensitivity scenarios where 100 percent of food waste is diverted from either WTE combustion or landfill disposal. In the Massachusetts disposal scenario 81 percent of collected landfill gas is used for energy recovery, while the rest is flared (Commonwealth of Massachusetts 2017b). In the national disposal mix scenario 68 percent of landfill gas is recovered for energy production, 24 percent is flared, and 8 percent is vented to the atmosphere (USA EPA 2017a, 2017b). Impact results for each scenario are presented relative to the baseline, which is represented as a black bar (at 0 percent) in Figure 3. The magnitude of impact results is sensitive to the presented avoided end-of-life options, as indicated by the spread of impact results within the partial and full capacity scenarios, respectively. All results in Figure 3 are for the base AD performance scenario. GCCP results are particularly sensitive to avoided end-of-life assumptions. The net GCCP impacts decrease 440–500 percent from the baseline if this facility is co-digesting food waste that would have been otherwise 100 percent sent to landfill. Alternatively, GCCP impacts from the baseline decrease 50 percent if this facility is co-digesting food waste that would have been otherwise 100 percent sent to WTE combustion. Overall, avoiding landfill disposal of food waste results in notably greater environmental benefits for both GCCP and CED than avoiding WTE combustion. However, the inclusion of different avoided food waste disposal options does not affect the down-ward trajectory of GCCP and CED impact results because the GLSD WWTF accepts SSO for co-digestion, as demonstrated by the position of all net co-digestion scenario results below the baseline in Figure 3. Although incorporating avoided food waste disposal within the system boundaries results in GCCP savings, the opposite is the case for CED. Because energy is produced from WTE combustion and landfill (through landfill gas recovery), excluding the avoided food waste disposal processes results in a net decrease in the CED results. However, the overall energy recovery benefits of co-digestion still outweigh energy benefits seen from other waste management options, such as WTE combustion.

**Figure 4 |:**
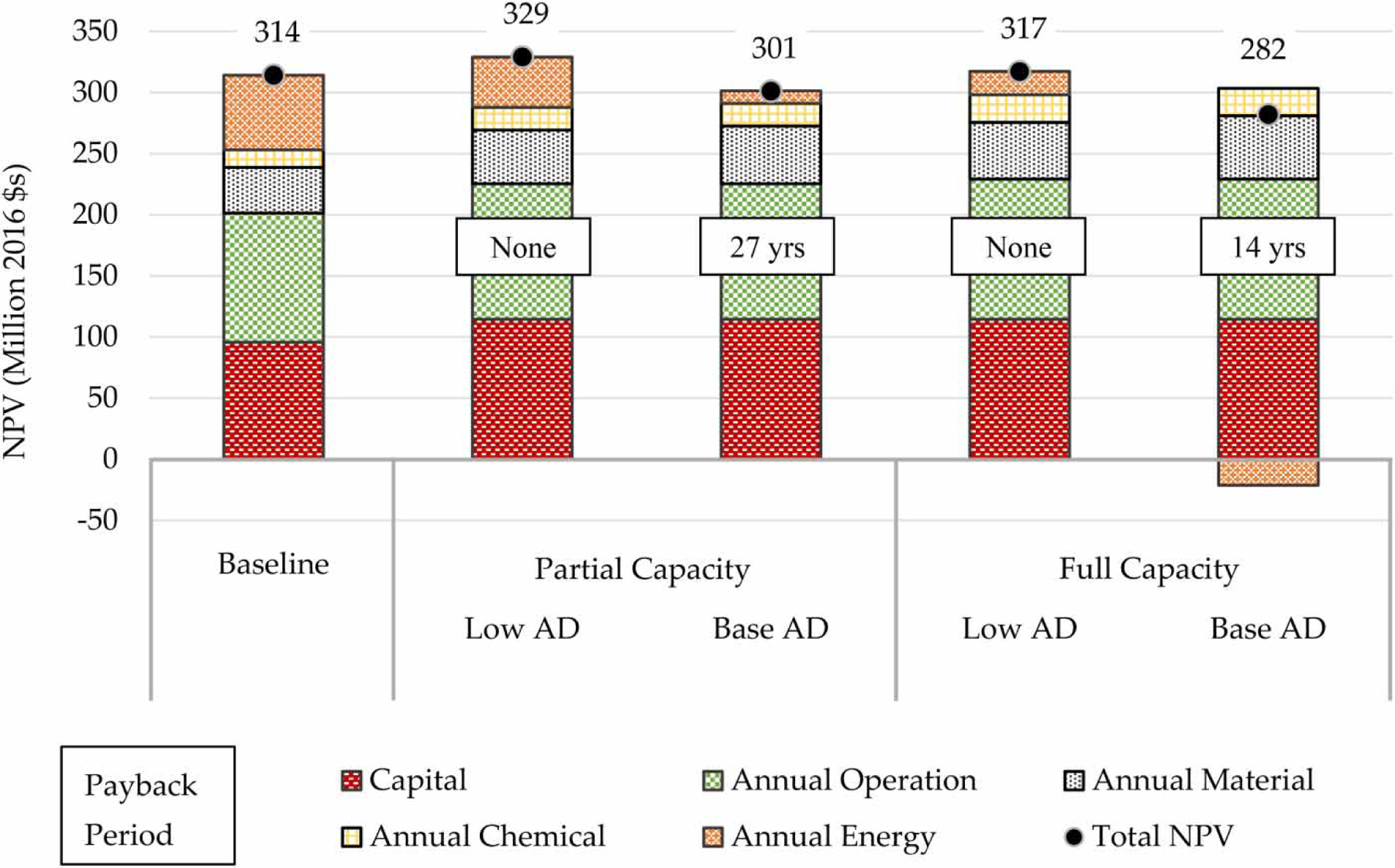
Base cost system NPV and discounted payback period of the GLSD Wastewater Treatment Facility AD and CHP expansion project. Results are shown for the low and base AD performance scenarios.

One benefit of the water resource recovery facility concept is the potential to generate additional sources of revenue that can supplement rate fees paid by domestic, institutional, and industrial customers. Installation of CHP and the acceptance of SSO gives the facility the opportunity to collect waste tipping fees for the SSO material and to sell renewable energy credits and alternative energy credits for the electrical and thermal energy generated by the CHP system. Although not a source of direct revenue, the net metering program gives energy generators the opportunity to avoid electricity costs, while maintaining a grid interconnection. Figure 4 presents system NPV and discounted payback period for the AD and CHP expansion project for each of the five LCA scenarios using the more conservative base cost assumptions. Total system NPV over the 30-year period is less than baseline scenario cost for the two base AD performance scenarios, corresponding to 14- and 27-year payback periods for the full and partial capacity scenarios, respectively. Reduced energy cost, or revenue in the case of the full capacity scenario, is responsible for system NPV reductions. SSO revenue minimizes increases in operational cost. Capital cost, annual material cost, and chemical costs all increase as a result of the AD and CHP expansion project. Table S11 provides complementary LCCA results for the low cost scenario.

## CONCLUSIONS

The magnitude of potential impact reductions and environmental benefits in six of the eight assessed environmental impact categories provides strong support for GLSD’s AD expansion and CHP project. The general trends observed identify municipal co-digestion and implementation of CHP systems as promising opportunities to avoid less desirable food waste disposal methods while reducing the environmental impact of the wastewater treatment sector. GCCP, CED, and FDP impact is reduced relative to the baseline scenario in all four SSO scenarios. AP, PMFP, and SFP impacts are reduced in all base AD performance SSO scenarios and increased marginally (<15 percent) in the low AD performance scenario. WU benefits are dominated by effluent reuse and are not considerably affected by SSO acceptance.

As demonstrated in Figure 2, EP is the only impact category for which impact results increase, regardless of the feedstock acceptance and AD performance scenario being considered. Given that the traditional purpose of WWTF is to protect aquatic ecosystems, this trade-off must be taken seriously. Although this poses no regulatory issues for the case-study facility, it will need to be carefully considered by other WWTF located in regions with more stringent effluent nutrient criteria. Monitoring data presented in Section S7 indicate that estimated increases in EP based on the GPS-X model results may be overestimated. However, additional monitoring data are required to verify this when the WWTF reaches full AD capacity. The surplus of energy produced in the full capacity-base AD performance scenario could also be used to enhance nutrient removal capacity through operational changes or investment. For facilities that are facing stringent effluent nutrient criteria, this energy surplus offers the opportunity to minimize or eliminate the environmental impact of increased electricity, chemical, and infrastructure demands required to increase nutrient removal.

The scenario and sensitivity results indicate that the opportunity to realize reduced impacts is remarkably robust. Considerable impact reductions are expected for CED, GCCP, and FDP across all co-digestion scenarios. In the base AD performance scenario, six of the eight impact categories studied yield net reductions in impact that exceed 35 percent, with eutrophication and water use being the only exception. Results generally show that environmental impacts are more sensitive to assumptions regarding AD performance than they are to capacity utilization. In the low AD performance scenario, AP and EP impact increases exceed 10 percent of potential baseline impacts, while increases in impact for SFP, PMFP, and WU are all negligible (i.e. <5 percent). Scenario results also indicate that greater GCCP savings are possible if this co-digestion strategy were to be implemented in regions that are currently primarily landfilling food waste.

This study reinforces previous work demonstrating the environmental benefits of AcD in a small-scale facility (Morelli *et al.* 2018) and indicates more favorable economic outcomes for medium-scale WWTF that invest in co-digestion, with the LCCA indicating payback periods of 14–27 years when base AD performance is achieved.

## Supplementary Material

Environmental and Cost Benefits of Co-Digesting Food Waste at Wastewater Treatment Plants SM

## Figures and Tables

**Table 1 |:** Feedstock scenario waste treatment volumes (m^3^/day)

Waste source	Baseline	Partial capacity	Full capacity
Primary and waste activated sludge	6.4 × 10^2^	6.8 × 10^2^	7.2 × 10^2^
Septage	3.0 × 10^2^	3.0 × 10^2^	3.0 × 10^2^
Trucked municipal solids^a^	30	30	30
SSO	–	1.7 × 10^2^	3.5 × 10^2^

a Trucked-in municipal solids refers to thickened primary and waste activated sludge from small, regional WWTFs.

**Table 2 |:** Anaerobic digester performance scenario parameters

Parameter name	Feedstock scenario	Low AD	Base AD
Percent VSR^a^ (% of influent VS)	Baseline	55%	
	Partial capacity	61%	69%
	Full capacity	63%	72%
Biogas yield^b^ (standard m^3^/kg of VS destroyed)	Baseline	1.1	
	Partial capacity	0.94	1.1
	Full capacity	0.94	1.2
Flaring rate^c^	All	20%	10%
Biogas production (m^3^/day)	Baseline	1.2 × 10^4^	
	Partial capacity	2.4 × 10^4^	3.3 × 10^4^
	Full capacity	3.8 × 10^4^	5.3 × 10^4^

a Low AD performance volatile solids reduction (VSR) assumes 50 percent for municipal solids, low end of range in Figure A-6 (Appleton & Rauch-Williams 2017), and 70 percent for SSO (11 percent reduction relative to Base AD VSR). VS, volatile solids.

b Biogas yield values for the base AD scenario were based on GPS-X model output (Hydromantis 2017). Low AD performance biogas yield was based on CAPDETWorks defaults (Harris et al. 1982).

c The baseline scenario has an 18-percent flaring rate, which was used as the basis of the low AD performance scenario, rounded to 20 percent. The base AD performance scenario assumes that the availability of CHP should reduce the facilities flaring rate.

**Table 3 |:** LCI by treatment process for liquid treatment processes (per m^3^ wastewater treated)^a^

					Partial capacity			Full capacity		
Treatment process	Inventory item	Units/m^3^	Baseline		Low AD	Base AD		Low AD	Base AD	
Preliminary and primary treatment	Electricity	kWh	0.202	★	0.202	0.202	★	0.202	0.202	★
	Activated carbon	kg	1.71 × 10^−4^	★	1.71 × 10^−4^	1.71 × 10^−4^	★	1.71 × 10^−4^	1.71 × 10^−4^	★
	Septage transport	tkm	0.095	★	0.095	0.095	★	0.095	0.095	★
	Grit disposal	kg	0.012	★	0.012	0.012	★	0.012	0.012	★
	Potassium permanganate	kg	4.62 × 10^−5^	★	4.62 × 10^−5^	4.62 × 10^−5^	★	4.62 × 10^−5^	4.62 × 10^−5^	★
Secondary treatment	Electricity	kWh	0.167	★	0.169	0.169	♦	0.172	0.172	♦
	Process emission, CH_4_	kg CH_4_	3.67 × 10^−3^	♦	3.68 × 10^−3^	3.68 × 10^−3^	♦	3.69 × 10^−3^	3.69 × 10^−3^	♦
	Process emission, N_2_O	kg N_2_O	9.52 × 10^−5^	♦	1.05 × 10^−4^	1.05 × 10^−4^	♦	1.12 × 10^−4^	1.12 × 10^−4^	♦
Plant water and disinfection	Electricity	kWh	0.029	★	0.029	0.029	★	0.029	0.029	★
	Sodium hypochlorite	kg	3.40 × 10^−3^	★	3.40 × 10^−3^	3.40 × 10^−3^	★	3.40 × 10^−3^	3.40 × 10^−3^	★
	Sodium bisulfite	kg	3.97 × 10^−3^	★	3.97 × 10^−3^	3.97×10^−3^	★	3.97 × 10^−3^	3.97 × 10^−3^	★
	Avoided potable water	m^3^	0.126	★	0.126	0.126	★	0.126	0.126	★
Effluent	Ammonia, to water	kg NH_3_	0.022	★	0.025	0.024	○	0.027	0.026	○
	Biological oxygen demand	kg O_2_	0.017	★	0.018	0.018	○	0.018	0.018	○
	Nitrate	kg NO_3_	2.80 × 10^−3^	★	3.14 × 10^−3^	3.06E-3	○	3.42 × 10^−3^	3.26 × 10^−3^	○
	Nitrogen, organic	kg N	1.41 × 10^−3^	★	1.58 × 10^−3^	1.54 × 10^−3^	○	1.73 × 10^−3^	1.65 × 10^−3^	○
	Phosphorus, total	kg P	3.61 × 10^−4^	★	3.81 × 10^−4^	3.73 × 10^−4^	○	4.02 × 10^−4^	3.84 × 10^−4^	○
	Suspended solids	kg TSS	5.95 × 10^−3^	★	6.20 × 10^−3^	6.20 × 10^−3^	○	6.48 × 10^−3^	6.48 × 10^−3^	○
	Nitrous oxide, to air	kg N_2_O	1.62 × 10^−4^	★	1.80 × 10^−4^	1.76 × 10^−4^	○	1.96 × 10^−4^	1.87 × 10^−4^	○
Operation, facilities	Electricity	kWh	7.24 × 10^−3^	★	7.60 × 10^−3^	7.60 × 10^−3^	★	7.60 × 10^−3^	7.60 × 10^−3^	★
	Natural gas	MJ	0.401	★	0.401	–	✚	–	–	✚
	Potable water	m^3^	4.52 × 10^−4^	★	4.52 × 10^−4^	4.52 × 10^−4^	★	4.52 × 10^−4^	4.52 × 10^−4^	★

a ★ – based on plant data, ○ – scaled baseline value, ✚ – allocated based on biogas use hierarchy, ♦ – based on GPS-X output.

AD – anaerobic digestion, tkm – ton kilometers, TSS – total suspended solids.

**Table 4 |:** LCI for solid treatment processes (per m^3^ wastewater treated)

					Partial capacity			Full capacity		
Treatment process	Inventory item	Units/m^3^	Baseline		Low AD	Base AD		Low AD	Base AD	
SSO processing	Transport	tkm	–	*	0.122	0.122	*	0.244	0.244	*
	Electricity	kWh	–	*	0.012	0.012	*	0.024	0.024	*
	Water	_m_3	–	*	1.19 × 10^−3^	1.19 × 10^−3^	*	2.40 ×10^−3^	2.40 × 10^−3^	*
Dewatering	Electricity	kWh	4.20 × 10^−3^	★	0.065	0.065	★	6.08 × 10^−3^	6.08 × 10^−3^	★
	Polymer	kg	4.20 × 10^−3^	★	6.08 × 10^−3^	6.08 × 10^−3^	○	7.92 × 10^−3^	7.92 × 10^−3^	○
Anaerobic digestion	Electricity	kWh	0.051	★	0.065	0.065	♦	0.065	0.065	♦
	Natural gas	MJ	0.123	★	0.134	–	✚	–	–	✚
	Ferric chloride	kg	5.93 × 10^−4^	★	6.67 × 10^−4^	6.67 × 10^−4^	○	7.42 × 10^−4^	7.42 × 10^−4^	○
	Process emission, CH_4_	kg CH_4_	2.56 × 10^−3^	♦	5.22 × 10^−3^	7.26 × 10^−3^	♦	8.43× 10^−3^	0.012	♦
	Biogas, to flare	m^3^	0.022	✚	0.051	0.035	✚	0.081	0.057	✚
	Biogas, to pellet drier	m^3^	0.067	✚	0.105	0.105	✚	0.142	0.142	✚
	Biogas, to glycol boiler	m^3^	0.036	✚	–	–	✚	–	–	✚
	Biogas, to CHP	m^3^	–	✚	0.098	0.213	✚	0.184	0.369	✚
	Avoided electricity	kWh	–	✚	0.226	0.490	✚	0.424	0.848	✚
	Avoided natural gas	MJ	2.10	✚	3.14	4.29	✚	4.74	6.58	✚
	Avoided landfill	kg SSO	–	●	0.661	0.661	●	1.32	1.32	●
	Avoided WTE	kg SSO	–	●	1.40	1.40	●	2.81	2.81	●
	Concrete	m^3^	–	□	7.37 × 10^−7^	7.37 × 10^−7^	□	7.37 × 10 ^−7^	7.37 × 10^−7^	□
	Excavation	m^3^	–	□	4.44 × 10^−6^	4.44 × 10^−6^	□	4.44 × 10^−6^	4.44 × 10^−6^	□
	Steel	kg	–	□	4.91 × 10^−5^	4.91 × 10^−5^	□	4.91 × 10^−5^	4.91 × 10^−5^	□
	Gravel	kg	–	□	3.60 × 10^−4^	3.60 × 10^−4^	□	3.60 × 10^−4^	3.60 × 10^−4^	□
	Building	m^3^	–	□	3.67 × 10^−6^	3.67 × 10^−6^	□	3.67 × 10^−6^	3.67 × 10^−6^	□
Pellet drying	Electricity	kWh	0.063	★	0.089	0.089	○	0.120	0.120	○
	Natural gas	MJ	0.033	★	0.022	–	✚	–	–	✚
Land application	Transport	tkm	0.019	▪	0.027	0.027	▪	0.037	0.037	▪
	Diesel, spreading	liters	1.69 × 10^−4^	▪	2.39 × 10^−4^	2.39 × 10^−4^	▪	.21 × 10^−4^	3.21 × 10^−4^	▪
	Avoided fertilizer, urea	kg N	3.50 × 10^−3^	▪	4.95 × 10^−3^	4.95 × 10^−3^	▪	6.66 × 10^−3^	6.66 × 10^−3^	▪
	Avoided fertilizer, single superphosphate	kg P_2_O_5_	3.03 × 10^−3^	▪	4.28 × 10^−3^	4.28 × 10^−3^	▪	5.75 × 10^−3^	5.75 × 10^−3^	▪
	Phosphorus, to water	kg P	6.23 × 10^−6^	▪	8.80 × 10^−6^	8.80 × 10^−6^	▪	1.18 × 10^−5^	1.18 × 10^−5^	▪
	Nitrate, to water	kg NO_3_	3.81 × 10^−3^	▪	5.38 × 10^−3^	5.38 × 10^−3^	▪	7.23 × 10^−3^	7.23 × 10^−3^	▪
	Ammonia, to air	kg NH_3_	2.96 × 10^−4^	▪	4.18 ×10 ^4^	4.18 × 10^−4^	▪	5.62 ×10^−4^	5.62 ×10^−4^	▪
	Nitrous oxide, to air	kg N_2_O	7.19 × 10^−5^	▪	1.02 × 10^−4^	1.02 × 10^−4^	▪	1.37 × 10^−4^	1.37 × 10^−4^	▪
	Carbon, sequestered	kg CO_2_	0.014	▪	0.020	0.020	▪	0.027	0.027	▪

a ★ – based on plant data, ○ – scaled baseline value, ● – based on Massachusetts disposal routes, ✚ – allocated based on biogas use hierarchy, □ – estimated based on unit dimensions, ♦ – based on GPS-X output, ▪ – calculated based on pellet characteristics, *– estimated using proxy assumptions.

AD – anaerobic digestion, CHP – combined heat and power, SSO – source separated organics, tkm – ton kilometers, WTE – waste to energy.

**Table 5 |:** Impact results summary (per m^3^ wastewater treated) and percent change from baseline LCA scenario

		Feedstock – AD performance scenario				
Impact category	Units	Baseline	Partial capacity – Base AD	Partial capacity – Low AD	Full capacity – Base AD	Full capacity – Low AD
Acidification potential	kg SO_2_ eq	1.0 × 10^−3^	6.6 × 10^−4^	1.1 × 10^−3^	5.4 × 10^−4^	1.1 × 10^−3^
	(% change)^a^	n.a.	−34%	13%	−46%	14%
Cumulative energy demand	MJ	5.0	−1.7	3.7	−6.4	1.2
	(% change)	n.a.	−134%	−27%	−226%	−76%
Eutrophication potential	kg N eq	0.02	0.03	0.03	0.03	0.03
	(% change)	n.a.	10%	14%	20%	24%
Fossil fuel depletion potential	kg oil eq	0.05	−0.07	0.02	−0.15	−0.04
	(% change)	n.a.	−248%	−60%	−430%	−184%
Global climate change potential	kg CO_2_ eq	0.36	0.01	0.19	−0.28	−0.05
	(% change)	n.a.	−98%	−47%	−176%	−113%
Particulate matter formation potential	kg PM_2.5_ eq	5.4 × 10^−5^	1.8 × 10^−5^	5.6 × 10^−5^	−4.5 × 10^−63^	4.4 × 10^−5^
	(% change)	n.a.	−67%	3%	−108%	−19%
Smog formation potential	kg O_3_ eq	0.02	8.3 × 10^−3^	0.02	3.7 × 10^−3^	0.02
	(% change)	n.a.	−50%	5%	−78%	2%
Water use	m^3^ H_2_O	0.13	−0.12	−0.12	−0.12	−0.12
	(% change)	n.a.	1%	1%	1%	2%

a Percentage change = [(Co-digestion impact Baseline impact)/Baseline impact].

**Table 6 |:** Annual facility energy demand summary

		Feedstock – AD performance scenario				
			Partial capacity		Full capacity	
Parameter	Units	Baseline	Base AD	Low AD	Base AD	Low AD
Total biogas energy	TJ	87.6	248	178	397	285
Loss, fugitive	TJ	4.38	12.4	8.91	19.8	14.2
Loss, flare	TJ	14.6	23.5	16.9	75.4	54.1
Use, boiler	TJ	24.1	–	–	–	–
Use, pellet dryer	TJ	44.4	70.0	70.0	94.1	94.1
Use, CHP electricity	TJ	–	57.2	26.4	98.9	49.4
Use, CHP thermal	TJ	–	49.9	31.8	57.1	57.1
Loss, CHP thermal	TJ	–	19.2	–	62.3	2.53
Electricity demand satisfied	%	–	80%	37%	100%^a^	64%
Thermal demand satisfied	%	79%	100%	85%	100%	100%
Total biogas energy satisfied	%	78%	81%	74%	71%	72%

a Exports 6.1 Gigawatt hours of excess electricity to the electrical grid annually.
