# Supplementary material for: Environmental and cost benefits of co-digesting food waste at wastewater treatment facilities
Source: Water Sci Technol. Author manuscript; Available in PMC 2021 Jul 1. (PMC7899238; doi:10.2166/wst.2020.104)
Supplement: Environmental and Cost Benefits of Co-Digesting Food Waste at Wastewater Treatment Plants SM [file NIHMS1659818-supplement-Environmental_and_Cost_Benefits_of_Co-Digesting_Food_Waste_at_Wastewater_Treatment_Plants_SM.docx]

Type of the Paper: Article (Research Paper)

Supplementary Materials

**Environmental and Cost Benefits of Co-Digesting Food Waste at Wastewater Treatment Facilities**

Ben Morelli^1^, Sarah Cashman^2^, Xin (Cissy) Ma^3,^*, Jason Turgeon^4^, Sam Arden^5^ and Jay Garland^6^

1. Eastern Research Group, 110 Hartwell Ave., Lexington, MA 02421, USA, [Ben.Morelli@erg.com](mailto:Ben.Morelli@erg.com)
2. Eastern Research Group, 110 Hartwell Ave., Lexington, MA 02421, USA, [Sarah.Cashman@erg.com](mailto:Sarah.Cashman@erg.com)

^3,^* United States Environmental Protection Agency, Center for Environmental Solutions and Emergency Response, Water Infrastructure Division, 26 West Martin Luther King Drive, Cincinnati, OH 45268, USA, [Ma.Cissy@epa.gov](mailto:Ma.Cissy@epa.gov), Tel.:+1-513-569-7828

^4^ United States Environmental Protection Agency, Region 1, 5 Post Office Square, Suite 100, OEP 5-2, Boston, MA 02109, [Turgeon.Jason@epa.gov](mailto:Turgeon.Jason@epa.gov)

^5^ Eastern Research Group, 110 Hartwell Ave., Lexington, MA 02421, USA, [Sam.Arden@erg.com](mailto:Sam.Arden@erg.com)

^6^ United States Environmental Protection Agency, Center for Environmental Solutions and Emergency Response, 26 West Martin Luther King Drive, Cincinnati, OH 45268, USA, [Garland.Jay@epa.gov](mailto:Garland.Jay@epa.gov)

***** Correspondence: Ma.Cissy@epa.gov; Tel.: +1-513-569-7828

1. Wastewater characterization

Table S1 lists average influent wastewater characteristics. Influent values serve as an input to the GPS-X™ model of the wastewater treatment facility (WWTF).

| Table S1. Average influent composition of case-study wastewater treatment facility | | |
| --- | --- | --- |
| Characteristic | Value | Unit |
| Total suspended solids (TSS) | 251 | mg/L |
| Volatile solids (VS) | 75% | – |
| Carbonaceous biological oxygen demand | 188 | mg/L |
| Total kjeldahl nitrogen | 35 | mg/L N |
| Ammonia (NH_3_) | 20 | mg/L N |
| Total phosphorus (TP) | 5.6 | mg/L P |
| Nitrite (NO_2_) | 0 | mg/L N |
| Nitrate (NO_3_) | 0 | mg/L N |
| Organic nitrogen | 15 | mg/L N |

Table Abbreviations: N – nitrogen, P - phosphorus.

1. Process greenhouse gas emissions

The analysis includes estimates of methane (CH_4_) and nitrous oxide (N_2_O) process greenhouse gas emissions from the biological treatment process based on IPCC guidelines for development of national greenhouse gas inventories (IPCC, 2006). Nitrous oxide emissions were estimated by applying an emission factor of 0.0016 kg N_2_O-N/kg of total kjeldahl nitrogen influent to the biological treatment process. Methane emissions were estimated using the biological oxygen demand concentrations influent to the biological treatment process. The IPCC method is based on theoretical maximum CH_4_ production (Bo) under completely anaerobic conditions, which equals 0.6 kg CH_4_/kg biological oxygen demand. Methane production is then scale downwards using a methane correction factor that reflects the presence of anaerobic conditions within the treatment process. A methane correction factor of 0.044 was used in this analysis based on values from (Daelman, van Voorthuizen, et al., 2013), associated with the operation of a Modified Ludzack-Ettinger biological treatment process. Results in the study indicate that 11 grams of CH_4_ are produced per kg of influent chemical oxygen demand. This emission factor was converted into a methane correction factor value using a Bo value of 0.25 kg CH_4_/kg chemical oxygen demand. The GPS-X™ model was used to estimate influent total kjeldahl nitrogen and biological oxygen demand concentrations in each of the four feedstock capacity-AD performance scenarios.

The study also accounts for N_2_O emissions that result from effluent discharge of nitrogen into receiving waters. An emission factor of 0.005 kg N_2_O-N/kg of effluent N was used to estimate secondary release of N_2_O from the aquatic environment (IPCC, 2006).

Five percent of produced CH_4_ was assumed to be lost from the AD tanks as fugitive emissions (UNFCCC, 2017).

1. Onsite combustion emissions

Biogas is burned at the WWTF in one of four combustion processes. Table S2 lists emissions to air associated with the combustion of one cubic meter of biogas in the flare, combined heat and power system (CHP), glycol boiler and pellet drier. Use of the glycol boiler is only associated with the baseline scenario, prior to the installation of the CHP system. Flare use is determined by the AD performance scenario and ranges from 10 to 20 percent of produced biogas. Use of biogas to fuel the pellet drier is prioritized over use of the CHP system.

| Table S2. Emission inventories used to estimate impact of onsite combustion processes | | | | |
| --- | --- | --- | --- | --- |
| **Pollutant** | **Flare** | **CHP** | **Glycol boiler** | **Pellet drier** |
|  | **Emissions (kg/m^3^ biogas combusted)** | | | |
| Ammonia (NH_3_) | n.a. | 6.41E-05 | n.a. | n.a. |
| Arsenic (As) | n.a. | n.a. | n.a. | 2.64E-08 |
| Cadmium (Cd) | n.a. | n.a. | n.a. | 1.66E-07 |
| Carbon monoxide (CO) | 3.47E-03 | 1.07E-03 | 2.40E-04 | 8.25E-04 |
| Methane (CH_4_), base AD performance^2^ | 3.90E-03 | 4.30E-03^1^ | 4.10E-05 | 4.10E-05 |
| Methane (CH_4_), low AD performance^2^ | 1.95E-02 |  |  |  |
| Nitrogen oxides (NOx) | 1.15E-03 | 1.64E-04 | 4.08E-04 | 1.12E-03 |
| Nitrous oxide (N_2_O) | n.a | 1.02E-04^1^ | 1.02E-05 | 2.05E-06 |
| Particulate matter (PM) | 5.81E-04 | 3.44E-05 | 1.20E-04 | 5.93E-04 |
| Sulfur dioxide (SO_2_) | 1.26E-03 | 1.41E-05 | 5.15E-04 | 1.21E-03 |
| Volatile organic compounds (VOCs), base AD performance^2^ | 8.07E-04 | 1.58E-03 | 9.29E-05 | 2.05E-04 |
| Volatile organic compounds (VOCs), low AD performance^2^ | 4.04E-03 |  |  |  |

^1^ Values are based on Ecoinvent 2.2 unit process: "natural gas, burned in cogen one MWe lean burn", and are converted to be on the basis of one cubic meter of biogas combustion (Ecoinvent, 2010, p. 2).

^2^ Methane and VOC emissions are based on a 99% and 95% destruction rates in the base and low AD performance scenarios, respectively.

Table Acronyms: AD – anaerobic digestion, CHP – combined heat and power.

1. Emission estimation from land applied biosolids pellets

Pelletized biosolids are land applied to agricultural fields in agricultural regions within a reasonable distance (121 km) of the WWTF. Table S3 lists the nutrient and carbon content of finished biosolids pellets at the point of transport and land application.

| Table S3. Biosolid pellet specifications | | |
| --- | --- | --- |
| **Parameter** | **Value** | **Units** |
| Moisture content | 2.5% | moisture |
| Nitrogen content | 4% | by weight |
| Phosphorus content | 2% | by weight |
| Potassium content | 0% | by weight |
| Carbon content | 28% | by weight |

Agricultural emissions resulting from pellet land application were calculated based on the fertilizer replacement rates reported in the main paper, assuming that the higher application rate of nitrogen and phosphorus necessary to achieve equivalent plant available nutrient applications will contribute additional emissions. Impact was estimated based on the net increase in agricultural emissions. This is a conservative approach, that may overestimate actual environmental impacts from replacing chemical fertilizers with pelletized biosolids. Table S4 lists emission factors used to develop the life cycle inventory (LCI).

| Table S4. Agricultural emission factors | | | |
| --- | --- | --- | --- |
| **Parameter** | **Value** | **Units** | **Source** |
| Carbon, sequestered | 0.32 | kg CO_2_/kg C applied | (Boldrin, Andersen, et al., 2009) |
| Ammonia, to air | 0.103 | kg NH_3_/kg applied N | (Goedkoop, Heijungs, et al., 2009) |
| Nitrogen oxides, to air^1^ | 0.011 | kg NO_x_/kg applied N | (Nemecek and Kägi, 2007) |
| Nitrate, to water | 1.33 | kg NO_3_/kg applied N | (De Klein, Novoa, et al., 2006) |
| Nitrous oxide, to air | 0.025 | kg N_2_O/kg applied N |  |
| Phosphorus, to groundwater | 2.99E-03 | kg P/kg applied P | (Nemecek and Kägi, 2007) |
| Phosphorus, to surface water | 0.087 | kg P/kg applied P |  |

Table Abbreviations: N – nitrogen, P - phosphorus.

1. Landfill and waste-to-energy life cycle inventory data

Table S5 lists LCI data for landfill disposal and waste-to-energy combustion of food waste. Inventories were developed based on national average and Massachusetts-based disposal statistics regarding treatment of landfill gas, as discussed in the main paper. Inventory data was developed using the U.S. EPA’s Municipal Solid Waste Decision Support Tool (MSW DST) (RTI International, 2012).

| Table S5. Life cycle inventory of national average and Massachusetts landfill disposal and waste-to-energy combustion | | | | | | |
| --- | --- | --- | --- | --- | --- | --- |
| **Parameter** | **Compartment** | **National average landfill** | **Massachusetts landfill** | **National Average WTE combustion** | **Massachusetts WTE combustion** | **Units** |
| Energy, diesel | input | 2.92E-07 | 2.92E-07 | 7.71E-02 | 7.71E-02 | MJ/kg SSO |
| Natural gas use | input | n.a. | n.a. | 2.00E-02 | 2.00E-02 | MJ/kg SSO |
| Electricity production | output | 0.025 | 0.021 | 8.70E-02 | 9.30E-02 | kWh/kg SSO |
| Total particulate matter | to air | 1.85E-05 | 1.65E-05 | 8.65E-06 | 1.16E-05 | kg/kg SSO |
| Nitrogen oxides | to air | 1.15E-04 | 1.03E-04 | 7.40E-05 | 1.67E-04 | kg/kg SSO |
| Hydrocarbons | to air | 7.00E-06 | 7.00E-06 | 1.86E-06 | 1.86E-06 | kg/kg SSO |
| Sulfur oxides | to air | 6.06E-06 | 5.97E-06 | 1.83E-05 | 3.14E-05 | kg/kg SSO |
| Carbon monoxide | to air | 3.43E-05 | 3.37E-05 | 1.75E-05 | 4.08E-05 | kg/kg SSO |
| Carbon dioxide biogenic | to air | 0.149 | 0.144 | 2.12E-01 | 2.12E-01 | kg/kg SSO |
| Carbon dioxide fossil | to air | 2.01E-03 | 2.01E-03 | 4.04E-03 | 4.04E-03 | kg/kg SSO |
| Ammonia | to air | 2.89E-09 | 2.89E-09 | 1.66E-06 | 1.66E-06 | kg/kg SSO |
| Lead | to air | 1.26E-11 | 1.26E-11 | 3.24E-09 | 3.24E-09 | kg/kg SSO |
| Methane | to air | 0.013 | 0.015 | 3.52E-06 | 3.52E-06 | kg/kg SSO |
| Hydrochloric acid | to air | 4.14E-06 | 3.83E-06 | 1.27E-05 | 1.27E-05 | kg/kg SSO |
| Dissolved solids | to water | 1.22E-05 | 1.22E-05 | 3.86E-06 | 3.86E-06 | kg/kg SSO |
| Suspended solids | to water | 3.10E-06 | 3.10E-06 | 1.42E-07 | 1.42E-07 | kg/kg SSO |
| BOD | to water | 1.45E-07 | 1.45E-07 | 5.92E-09 | 5.92E-09 | kg/kg SSO |
| COD | to water | 2.77E-08 | 2.77E-08 | 2.45E-07 | 2.45E-07 | kg/kg SSO |
| Oil | to water | 2.10E-07 | 2.10E-07 | 4.37E-07 | 4.37E-07 | kg/kg SSO |
| Sulfuric acid | to water | 4.20E-05 | 4.20E-05 | 1.16E-08 | 1.16E-08 | kg/kg SSO |
| Iron | to water | 6.17E-10 | 6.17E-10 | 6.26E-08 | 6.26E-08 | kg/kg SSO |
| Ammonia | to water | 1.74E-09 | 1.74E-09 | 4.32E-08 | 4.32E-08 | kg/kg SSO |
| Copper | to water | 3.55E-09 | 3.55E-09 | 2.83E-14 | 2.83E-14 | kg/kg SSO |
| Cadmium | to water | 2.69E-14 | 2.69E-14 | 1.76E-10 | 1.76E-10 | kg/kg SSO |
| Arsenic | to water | 1.16E-10 | 1.16E-10 | 7.87E-14 | 7.87E-14 | kg/kg SSO |
| Mercury | to water | 1.05E-14 | 1.05E-14 | 1.35E-14 | 1.35E-14 | kg/kg SSO |
| Phosphate | to water | 8.76E-15 | 8.76E-15 | 5.80E-09 | 5.80E-09 | kg/kg SSO |
| Selenium | to water | 3.19E-10 | 3.19E-10 | 1.69E-13 | 1.69E-13 | kg/kg SSO |
| Chromium | to water | 2.74E-14 | 2.74E-14 | 1.71E-10 | 1.71E-10 | kg/kg SSO |
| Lead | to water | 1.16E-10 | 1.16E-10 | 1.66E-12 | 1.66E-12 | kg/kg SSO |
| Zinc | to water | 1.35E-12 | 1.35E-12 | 6.29E-11 | 6.29E-11 | kg/kg SSO |

Table Acronyms: BOD – biological oxygen demand, COD – chemical oxygen demand, SSO – source separated organics, WTE – waste-to-energy.

1. Life cycle cost analysis

Table S6 lists scheduled debt service payments provided by the treatment plant for implemented and scheduled projects. The life cycle cost analysis (LCCA) carries the first ten years of baseline debt service payments into the future assuming that additional projects will be identified and carried out at a level that is equivalent to the period between 2016 and 2025.

| Table S6. Reported and modeled debt service | | | | | | |
| --- | --- | --- | --- | --- | --- | --- |
| **Analysis year** | **Year** | **Debt service ($ 2016) - plant records** | | | **Debt service ($ 2016) - analysis values** | |
|  |  | **Baseline** | **CHP-AD Project** | **Partial and full capacity** | **Baseline** | **Partial and full capacity** |
| year 1 | 2016 | $ 3,316,494 | $ - | $3,316,494 | $ 3,316,494 | $3,316,494 |
| year 2 | 2017 | $ 3,376,809 | $ - | $3,376,809 | $ 3,376,809 | $3,376,809 |
| year 3 | 2018 | $ 3,255,369 | $ - | $3,255,369 | $ 3,255,369 | $3,255,369 |
| year 4 | 2019 | $ 3,133,929 | $ - | $3,133,929 | $ 3,133,929 | $3,133,929 |
| year 5 | 2020 | $ 3,137,225 | $ - | $3,137,225 | $ 3,137,225 | $3,137,225 |
| year 6 | 2021 | $ 2,651,634 | $1,427,317 | $4,078,951 | $ 2,651,634 | $4,078,951 |
| year 7 | 2022 | $ 2,630,064 | $1,409,541 | $4,039,605 | $ 2,630,064 | $4,039,605 |
| year 8 | 2023 | $ 2,630,652 | $1,411,210 | $4,041,862 | $ 2,630,652 | $4,041,862 |
| year 9 | 2024 | $ 2,583,593 | $1,412,916 | $3,996,508 | $ 2,583,593 | $3,996,508 |
| year 10 | 2025 | $ 2,580,475 | $1,414,657 | $3,995,132 | $ 2,580,475 | $3,995,132 |
| year 11 | 2026 | $ 2,577,294 | $1,416,437 | $3,993,730 | $ 2,929,625 | $4,346,061 |
| year 12 | 2027 | $ 1,302,075 | $1,418,256 | $2,720,331 | $ 2,929,625 | $4,347,881 |
| year 13 | 2028 | $ 1,300,708 | $1,420,114 | $2,720,822 | $ 2,929,625 | $4,349,739 |
| year 14 | 2029 | $ 1,299,311 | $1,422,013 | $2,721,324 | $ 2,929,625 | $4,351,637 |
| year 15 | 2030 | $ 1,297,883 | $1,423,952 | $2,721,835 | $ 2,929,625 | $4,353,577 |
| year 16 | 2031 | $ 1,296,425 | $1,425,934 | $2,722,359 | $ 2,929,625 | $4,355,559 |
| year 17 | 2032 | $ 1,294,934 | $1,427,960 | $2,722,893 | $ 2,929,625 | $4,357,584 |
| year 18 | 2033 | $ 1,293,412 | $1,430,028 | $2,723,440 | $ 2,929,625 | $4,359,653 |
| year 19 | 2034 | $ 712,567 | $1,432,143 | $2,144,709 | $ 2,929,625 | $4,361,767 |
| year 20 | 2035 | $ 710,842 | $1,434,302 | $2,145,144 | $ 2,929,625 | $4,363,927 |
| year 21 | 2036 | $ 553,055 | $1,436,509 | $1,989,565 | $ 2,929,625 | $4,366,134 |
| year 22 | 2037 | $ 551,218 | $1,438,764 | $1,989,982 | $ 2,929,625 | $4,368,389 |
| year 23 | 2038 | $ 549,341 | $1,441,068 | $1,990,409 | $ 2,929,625 | $4,370,693 |
| year 24 | 2039 | $ 547,424 | $1,443,422 | $1,990,846 | $ 2,929,625 | $4,373,047 |
| year 25 | 2040 | $ 545,464 | $1,445,828 | $1,991,292 | $ 2,929,625 | $4,375,452 |
| year 26 | 2041 | $ 1,805,128 | $ - | $2,946,423 | $ 2,929,625 | $2,929,625 |
| year 27 | 2042 | $ 1,805,128 | $ - | $2,946,423 | $ 2,929,625 | $2,929,625 |
| year 28 | 2043 | $ 1,805,128 | $ - | $2,946,423 | $ 2,929,625 | $2,929,625 |
| year 29 | 2044 | $ 1,805,128 | $ - | $2,946,423 | $ 2,929,625 | $2,929,625 |
| year 30 | 2045 | $ 1,805,128 | $ - | $2,946,423 | $ 2,929,625 | $2,929,625 |

Table Acronyms: AD – anaerobic digestion, CHP – combined heat and power.

Table S7 lists the energy cost escalation factors used in the LCCA (Lavappa, Kneifel, et al., 2017). Energy costs are the only prices that are escalated beyond the standard rate of inflation.

| Table S7. Energy cost escalation factors | | | |
| --- | --- | --- | --- |
| **Year** | **Distillate fuel oil escalation factor** | **Natural gas escalation factor^1^** | **Electricity escalation factor^1^** |
| 2016 | 1.00 | 1.00 | 1.00 |
| 2017 | 1.00 | 1.00 | 1.00 |
| 2018 | 1.12 | 1.02 | 0.980 |
| 2019 | 1.19 | 1.08 | 0.990 |
| 2020 | 1.22 | 1.18 | 1.01 |
| 2021 | 1.23 | 1.23 | 1.01 |
| 2022 | 1.25 | 1.26 | 1.03 |
| 2023 | 1.27 | 1.28 | 1.04 |
| 2024 | 1.29 | 1.30 | 1.05 |
| 2025 | 1.32 | 1.30 | 1.07 |
| 2026 | 1.34 | 1.29 | 1.09 |
| 2027 | 1.36 | 1.29 | 1.10 |
| 2028 | 1.37 | 1.31 | 1.11 |
| 2029 | 1.38 | 1.33 | 1.13 |
| 2030 | 1.41 | 1.35 | 1.14 |
| 2031 | 1.44 | 1.35 | 1.14 |
| 2032 | 1.47 | 1.36 | 1.14 |
| 2033 | 1.47 | 1.36 | 1.14 |
| 2034 | 1.49 | 1.38 | 1.13 |
| 2035 | 1.50 | 1.42 | 1.14 |
| 2036 | 1.53 | 1.45 | 1.15 |
| 2037 | 1.54 | 1.47 | 1.15 |
| 2038 | 1.55 | 1.48 | 1.15 |
| 2039 | 1.56 | 1.50 | 1.15 |
| 2040 | 1.58 | 1.49 | 1.15 |
| 2041 | 1.58 | 1.49 | 1.14 |
| 2042 | 1.58 | 1.50 | 1.14 |
| 2043 | 1.58 | 1.51 | 1.15 |
| 2044 | 1.58 | 1.53 | 1.15 |
| 2045 | 1.59 | 1.56 | 1.15 |

Table S8 lists the parameter values used to define the base and low cost scenarios. Only base cost results are presented in the main manuscript. Parameter values used in the low cost scenario lead to lower estimates of system net present value (NPV) and are more optimistic about the revenue potential of energy credits and waste tipping fees.

| Table S8. Base and low cost scenario parameter values | | | |
| --- | --- | --- | --- |
| **Parameter value** | **Low cost** | **Base cost** |  |
| Planning period (years) | 30 | 30 |  |
| Real discount rate (%) | 5% | 3% |  |
| Electricity cost ($/kWh)^1^ | 0.143 | 0.143 |  |
| Electricity, avoided cost ($/kWh)^2^ | 0.129 | 0.123 |  |
| Renewable energy credit ($/MWh)^3^ | 25 | 12 |  |
| Alternative energy credit ($/MWh)^3^ | 20 | 14 |  |
| Natural gas cost ($/DTH)^4^ | 10.5 | 9.88 |  |
| SSO tipping fee ($/gallon) | 0.02 | 0.005 |  |

^1^ 2016 plant utility bills, includes all fees.

^2^ Low cost value based on the energy feasibility study (CDM Smith, 2013). Base value assumes a 10% reduction in the 2016 utility rate to account for customer charges and system benefit not offset by net metering.

^3^- Low cost renewable energy credit (REC) and alternative energy credit (AEC) values based on personal communication with the program manager of Massachusetts’s Renewable Energy Portfolio Standard and Alternative Energy Portfolio Standard (Wassam, 2018). Base cost REC and AEC values based on correspondence with Greater Lawrence Sanitary District staff.

^4^ Base cost from plant utility records, Low scenario cost (PES and UTS, 2009)

Table Acronym: DTH – dekatherm = 1,000,000 British thermal units, SSO – source separated organics.

1. Plant effluent monitoring data

Over the course of this research, the facility began to accept source separated organics (SSO) for co-digestion with municipal solids. Figure S1, Figure S2 and Figure S3 plot monitoring data for nitrate/nitrite, ammonia and total phosphorus before and after SSO acceptance begins. The red vertical line in each figure corresponds to the start of SSO acceptance. Table S9 presents the results of a sample t-test performed on the available effluent data, which indicates no significant change in the concentration of nutrient pollutants leaving the WWTF.


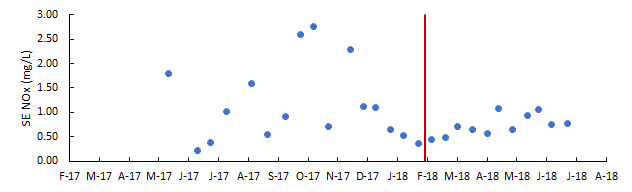


Figure S1. Effluent nitrate/nitrite concentrations from February 2017 to June 2018.


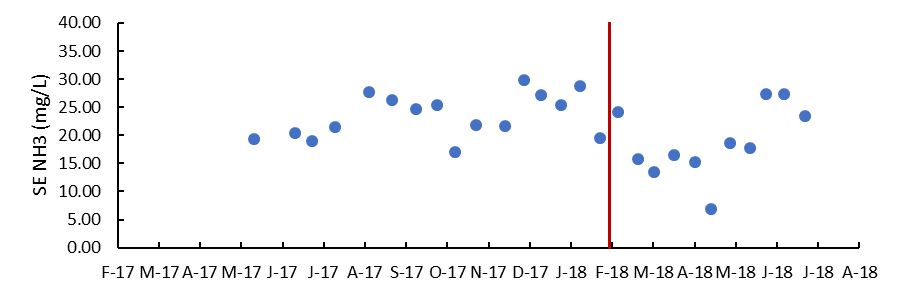


Figure S2. Effluent ammonia concentrations from February 2017 to June 2018.


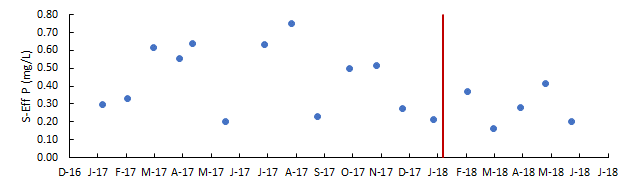


Figure S3. Effluent total phosphorus concentrations from February 2017 to June 2018.

| Table S9. Effluent sample *t*-test results | | | | | | |
| --- | --- | --- | --- | --- | --- | --- |
| **Effluent parameter** | **Months** |  | **2017 Mean^1^** | **2018 Mean^1^** | **Change** | ***P*-value** |
| NOx | May-Jun |  | 0.795 (3) | 0.872 (4) | 10% | 0.89 |
| NH₃ | May-Jun |  | 19.6 (3) | 24.0 (4) | 23% | 0.16 |
| TP | Feb-Jun |  | 0.469 (5) | 0.285 (5) | -39% | 0.11 |

^1^ Parenthesis value refers to the number of samples.

1. Life cycle assessment process results

Table S10 lists LCA results for the Massachusetts’s disposal mix scenario according to the treatment processes contributing impact. Results are presented for eight environmental impact categories.

| Table S10. Process LCIA results for the MA disposal mix avoided source separated organics disposal scenario  (per m^3^ wastewater treated) | | | | | | |
| --- | --- | --- | --- | --- | --- | --- |
|  | **AD Performance Scenario** | **Base** | **Base** | **Low** | **Base** | **Low** |
|  | **Feedstock scenario** | **Baseline** | **Partial capacity** | **Partial capacity** | **Full capacity** | **Full capacity** |
| Global warming potential - kg CO_2_ eq | WWTF, total | 0.36 | 6.6E-3 | 0.19 | -0.28 | -0.05 |
|  | Land application | -3.7E-3 | -5.4E-3 | -5.4E-3 | -7.3E-3 | -7.3E-3 |
|  | Preliminary/primary | 0.03 | 0.03 | 0.03 | 0.03 | 0.03 |
|  | Pellet drying | 0.03 | 0.04 | 0.04 | 0.05 | 0.05 |
|  | Influent pump station | 0.07 | 0.07 | 0.07 | 0.07 | 0.07 |
|  | Biological treatment | 0.18 | 0.18 | 0.18 | 0.18 | 0.18 |
|  | Sludge dewatering | 0.03 | 0.04 | 0.04 | 0.05 | 0.05 |
|  | Plant water and disinfection | -0.02 | -0.02 | -0.02 | -0.02 | -0.02 |
|  | Building operation | 0.03 | 3.3E-3 | 0.03 | 3.3E-3 | 3.3E-3 |
|  | Secondary clarification | 0.01 | 0.01 | 0.01 | 0.01 | 0.01 |
|  | Effluent release | 0.05 | 0.05 | 0.05 | 0.06 | 0.06 |
|  | Anaerobic digestion and CHP | -0.05 | -0.40 | -0.24 | -0.71 | -0.48 |
| Eutrophication potential - kg N eq | WWTF, total | 0.02 | 0.03 | 0.03 | 0.03 | 0.03 |
|  | Land application | 9.0E-4 | 1.3E-3 | 1.3E-3 | 1.7E-3 | 1.7E-3 |
|  | Preliminary/primary | 2.8E-5 | 2.8E-5 | 2.8E-5 | 2.8E-5 | 2.8E-5 |
|  | Pellet drying | 6.3E-6 | 9.0E-6 | 9.2E-6 | 1.2E-5 | 1.2E-5 |
|  | Influent pump station | 7.4E-6 | 7.4E-6 | 7.4E-6 | 7.4E-6 | 7.4E-6 |
|  | Biological treatment | 5.6E-6 | 5.7E-6 | 5.7E-6 | 5.8E-6 | 5.8E-6 |
|  | Sludge dewatering | 5.9E-5 | 8.6E-5 | 8.6E-5 | 1.1E-4 | 1.1E-4 |
|  | Plant water and disinfection | -2.9E-5 | -2.9E-5 | -2.9E-5 | -2.9E-5 | -2.9E-5 |
|  | Building operation | 4.7E-6 | 5.6E-7 | 4.7E-6 | 5.6E-7 | 5.6E-7 |
|  | Secondary clarification | 1.4E-6 | 1.4E-6 | 1.4E-6 | 1.4E-6 | 1.4E-6 |
|  | Effluent release | 0.02 | 0.02 | 0.02 | 0.03 | 0.03 |
|  | Anaerobic digestion and CHP | -1.3E-5 | -1.9E-5 | 4.9E-6 | -5.0E-5 | -1.5E-5 |
| Cumulative Energy Demand - MJ | WWTF, total | 5.0 | -1.7 | 3.7 | -6.4 | 1.2 |
|  | Land application | -0.23 | -0.32 | -0.32 | -0.43 | -0.43 |
|  | Preliminary/primary | 1.1 | 1.1 | 1.1 | 1.1 | 1.1 |
|  | Pellet drying | 0.83 | 1.1 | 1.1 | 1.5 | 1.5 |
|  | Influent pump station | 2.2 | 2.2 | 2.2 | 2.2 | 2.2 |
|  | Biological treatment | 1.7 | 1.7 | 1.7 | 1.7 | 1.7 |
|  | Sludge dewatering | 0.83 | 1.1 | 1.1 | 1.4 | 1.4 |
|  | Plant water and disinfection | -0.62 | -0.62 | -0.62 | -0.62 | -0.62 |
|  | Building operation | 0.58 | 0.10 | 0.59 | 0.10 | 0.10 |
|  | Secondary clarification | 0.41 | 0.41 | 0.41 | 0.41 | 0.41 |
|  | Effluent release | - | - | - | - | - |
|  | Anaerobic digestion and CHP | -1.8 | -8.5 | -3.6 | -14 | -6.2 |
| Fossil Depletion Potential - kg oil eq | WWTF, total | 0.05 | -0.07 | 0.02 | -0.15 | -0.04 |
|  | Land application | -4.1E-3 | -5.9E-3 | -5.9E-3 | -7.9E-3 | -7.9E-3 |
|  | Preliminary/primary | 0.02 | 0.02 | 0.02 | 0.02 | 0.02 |
|  | Pellet drying | 0.01 | 0.01 | 0.01 | 0.02 | 0.02 |
|  | Influent pump station | 0.03 | 0.03 | 0.03 | 0.03 | 0.03 |
|  | Biological treatment | 0.02 | 0.02 | 0.02 | 0.02 | 0.02 |
|  | Sludge dewatering | 0.01 | 0.02 | 0.02 | 0.02 | 0.02 |
|  | Plant water and disinfection | -7.5E-3 | -7.5E-3 | -7.5E-3 | -7.5E-3 | -7.5E-3 |
|  | Building operation | 0.01 | 1.2E-3 | 0.01 | 1.2E-3 | 1.2E-3 |
|  | Secondary clarification | 4.9E-3 | 4.9E-3 | 4.9E-3 | 4.9E-3 | 4.9E-3 |
|  | Effluent release | - | - | - | - | - |
|  | Anaerobic digestion and CHP | -0.05 | -0.16 | -0.08 | -0.25 | -0.14 |
| Particulate Matter Formation Potential - kg PM_2.5_ eq | WWTF, total | 5.4E-5 | 1.8E-5 | 5.6E-5 | -4.5E-6 | 4.4E-5 |
|  | Land application | 1.7E-6 | 2.3E-6 | 2.3E-6 | 3.2E-6 | 3.2E-6 |
|  | Preliminary/primary | 1.6E-5 | 1.6E-5 | 1.6E-5 | 1.6E-5 | 1.6E-5 |
|  | Pellet drying | 1.8E-5 | 2.7E-5 | 2.8E-5 | 3.7E-5 | 3.7E-5 |
|  | Influent pump station | 9.1E-6 | 9.1E-6 | 9.1E-6 | 9.1E-6 | 9.1E-6 |
|  | Biological treatment | 6.8E-6 | 6.9E-6 | 6.9E-6 | 7.0E-6 | 7.0E-6 |
|  | Sludge dewatering | 1.1E-5 | 1.5E-5 | 1.5E-5 | 1.9E-5 | 1.9E-5 |
|  | Plant water and disinfection | 7.1E-8 | 7.1E-8 | 7.1E-8 | 7.1E-8 | 7.1E-8 |
|  | Building operation | 5.7E-6 | 4.0E-7 | 5.7E-6 | 4.0E-7 | 4.0E-7 |
|  | Secondary clarification | 1.7E-6 | 1.7E-6 | 1.7E-6 | 1.7E-6 | 1.7E-6 |
|  | Effluent release | - | - | - | - | - |
|  | Anaerobic digestion and CHP | -1.6E-5 | -6.1E-5 | -2.9E-5 | -9.8E-5 | -5.0E-5 |
| Acidification Potential - kg SO_2_ eq | WWTF, total | 1.0E-3 | 6.6E-4 | 1.1E-3 | 5.4E-4 | 1.1E-3 |
|  | Land application | 4.1E-4 | 5.8E-4 | 5.8E-4 | 7.8E-4 | 7.8E-4 |
|  | Preliminary/primary | 1.8E-4 | 1.8E-4 | 1.8E-4 | 1.8E-4 | 1.8E-4 |
|  | Pellet drying | 1.9E-4 | 2.8E-4 | 2.8E-4 | 3.7E-4 | 3.7E-4 |
|  | Influent pump station | 1.3E-4 | 1.3E-4 | 1.3E-4 | 1.3E-4 | 1.3E-4 |
|  | Biological treatment | 1.0E-4 | 1.0E-4 | 1.0E-4 | 1.0E-4 | 1.0E-4 |
|  | Sludge dewatering | 9.6E-5 | 1.3E-4 | 1.3E-4 | 1.7E-4 | 1.7E-4 |
|  | Plant water and disinfection | -6.3E-6 | -6.3E-6 | -6.3E-6 | -6.3E-6 | -6.3E-6 |
|  | Building operation | 6.7E-5 | 6.0E-6 | 6.7E-5 | 6.0E-6 | 6.0E-6 |
|  | Secondary clarification | 2.5E-5 | 2.5E-5 | 2.5E-5 | 2.5E-5 | 2.5E-5 |
|  | Effluent release | - | - | - | - | - |
|  | Anaerobic digestion and CHP | -1.8E-4 | -7.6E-4 | -3.6E-4 | -1.2E-3 | -6.2E-4 |
| Smog Formation Potential - kg O_3_ eq | WWTF, Total | 0.02 | 8.3E-3 | 0.02 | 3.7E-3 | 0.02 |
|  | Land application | -3.0E-4 | -4.3E-4 | -4.3E-4 | -5.5E-4 | -5.5E-4 |
|  | Preliminary/primary | 3.6E-3 | 3.6E-3 | 3.6E-3 | 3.6E-3 | 3.6E-3 |
|  | Pellet drying | 3.6E-3 | 5.3E-3 | 5.4E-3 | 7.2E-3 | 7.2E-3 |
|  | Influent pump station | 4.6E-3 | 4.6E-3 | 4.6E-3 | 4.6E-3 | 4.6E-3 |
|  | Biological treatment | 3.6E-3 | 3.6E-3 | 3.6E-3 | 3.7E-3 | 3.7E-3 |
|  | Sludge dewatering | 1.6E-3 | 2.1E-3 | 2.1E-3 | 2.5E-3 | 2.5E-3 |
|  | Plant water and disinfection | -1.1E-3 | -1.1E-3 | -1.1E-3 | -1.1E-3 | -1.1E-3 |
|  | Building operation | 7.6E-4 | 2.1E-4 | 7.7E-4 | 2.1E-4 | 2.1E-4 |
|  | Secondary clarification | 8.6E-4 | 8.6E-4 | 8.6E-4 | 8.6E-4 | 8.6E-4 |
|  | Effluent release | - | - | - | - | - |
|  | Anaerobic digestion and CHP | -3.0E-4 | -0.01 | -1.7E-3 | -0.02 | -3.8E-3 |
| Water Use - m^3^ H_2_O | WWTF, Total | -0.13 | -0.12 | -0.12 | -0.12 | -0.12 |
|  | Land application | -3.4E-4 | -4.7E-4 | -4.7E-4 | -6.4E-4 | -6.4E-4 |
|  | Preliminary/primary | 8.4E-5 | 8.4E-5 | 8.4E-5 | 8.4E-5 | 8.4E-5 |
|  | Pellet drying | 8.8E-5 | 1.2E-4 | 1.2E-4 | 1.7E-4 | 1.7E-4 |
|  | Influent pump station | 2.5E-4 | 2.5E-4 | 2.5E-4 | 2.5E-4 | 2.5E-4 |
|  | Biological treatment | 1.9E-4 | 1.9E-4 | 1.9E-4 | 1.9E-4 | 1.9E-4 |
|  | Sludge dewatering | 1.1E-4 | 1.4E-4 | 1.4E-4 | 1.8E-4 | 1.8E-4 |
|  | Plant water and disinfection | -0.13 | -0.13 | -0.13 | -0.13 | -0.13 |
|  | Building operation | 4.7E-4 | 4.6E-4 | 4.7E-4 | 4.6E-4 | 4.6E-4 |
|  | Secondary clarification | 4.6E-5 | 4.6E-5 | 4.6E-5 | 4.6E-5 | 4.6E-5 |
|  | Effluent release | - | - | - | - | - |
|  | Anaerobic Digestion and CHP | 7.0E-5 | 8.3E-4 | 1.2E-3 | 1.7E-3 | 2.3E-3 |

Table Acronyms: AD – anaerobic digestion, CHP – combined heat and power, WWTF – wastewater treatment facility

1. Life cycle cost analysis – low cost scenario results

Table S11 lists LCCA summary results by cost category for the base and low cost scenarios. Base cost results correspond to Figure 4 in the main paper.

| Table S11. Life cycle cost analysis results – base and low cost scenario  (NPV in million 2016 $s) | | | | | | | |
| --- | --- | --- | --- | --- | --- | --- | --- |
| **Cost scenario** | **Scenario^1^** | **Capital** | **Annual operation** | **Annual material** | **Annual chemical** | **Annual energy** | **Total NPV** |
| Base Cost | Baseline | 96 | 106 | 37 | 14 | 61 | 314 |
|  | Partial Capacity  -Low AD | 115 | 111 | 44 | 18 | 41 | 329 |
|  | Partial Capacity  -Base AD | 115 | 111 | 47 | 18 | 10 | 301 |
|  | Full Capacity  -Low AD | 115 | 115 | 46 | 23 | 19 | 317 |
|  | Full Capacity  -Base AD | 115 | 115 | 52 | 23 | (21) | 282 |
| Low Cost | Baseline | 77 | 85 | 30 | 11 | 49 | 251 |
|  | Partial Capacity  -Low AD | 91 | 85 | 35 | 15 | 29 | 255 |
|  | Partial Capacity  -Base AD | 91 | 85 | 38 | 15 | 2 | 230 |
|  | Full Capacity  -Low AD | 91 | 84 | 37 | 18 | 9 | 239 |
|  | Full Capacity  -Base AD | 91 | 84 | 41 | 18 | (28) | 207 |

^1^ Feedstock capacity scenario-anaerobic digester performance scenario.

Table Acronyms: AD – anaerobic digestion, NPV – net present value.

1. References

Boldrin, A., Andersen, J. K., Møller, Jacob, Thomas, H., & Favoino, E. 2009 Composting and compost utilization: accounting of greenhouse gases and global warming contributions. *Waste Management and Research*, **27**(8), 800–812

CDM Smith. 2013 *Technical Memorandum Report: Greater Lawrence Sanitary District, Organics to Energy Feasibility Study*.

Daelman, M. R., van Voorthuizen, E. M., van Dongen, L. G., Volcke, El., & van Loosdrecht, M. C. 2013 Methane and Nitrous Oxide Emissions from Municipal Wastewater Treatment–Results from a Long-Term Study. *Water Science Technology*, **67**(10), 2350–2355.

De Klein, C., Novoa, R. S. A., Ogle, S., Smith, K. A., Rochette, P., & Wirth, T. C. 2006 *N2O Emissions from Managed Soils, and CO_2_ Emissions from Lime and Urea Application*, Intergovernmental Panel on Climate Change, National Greenhouse Gas Inventories Programme, IGES, Japan.

Ecoinvent. 2010 *Ecoinvent data version 2.2*. [online] http://www.ecoinvent.org/ (Accessed June 7, 2017).

Goedkoop, M., Heijungs, R., Huijbregts, M., De Schryver, A., Struijs, J., & van Zelm, R. 2009 ReCiPe 2008 *A life cycle impact assessment method which comprises harmonised category indicators at the midpoint and the endpoint level: Report 1 Characterization*, [online] http://www.leidenuniv.nl/cml/ssp/publications/recipe_characterisation.pdf (Accessed August 1, 2019).

IPCC. 2006 *IPCC Guidelines for National Greenhouse Gas Inventories*, Intergovernmental Panel on Climate Change, National Greenhouse Gas Inventories Programme, IGES, Japan.

Lavappa, P. D., Kneifel, J. D., & O’Rear, E. 2017 *Energy Price Indices and Discount Factors for Life-Cycle Cost Analysis – 2017 Annual Supplement to NIST Handbook 135*, National Institute of Standards and Technology.

Nemecek, T. & Kägi, T. 2007 *Life Cycle Inventories of Agricultural Production Systems*, Ecoinvent Centre, Dübendorf and Zürich, Switzerland. https://db.ecoinvent.org/reports/15_Agriculture.pdf (Accessed February 1, 2019).

PES & UTS. 2009 *Energy Evaluation for the Greater Lawrence Sanitary District Wastewater Treatment Facility and Riverside Pump Station*, Process Energy Services, LLC and UTS Energy Engineering, LLC.

RTI International. 2012 Municipal Solid Waste Decision Support Tool, RTI International. [online] https://mswdst.rti.org/ (Accessed August 1, 2019).

UNFCCC. 2017 *Methodological Tool, Project and leakage emissions from anaerobic digestion*. Report Tool14, version 02.0. United Nations Framework Convention on Climate Change.

Wassam, J. 2018 Personal Communication with John Wassam, RPS and APS Program Manager.
